# Evidence-informed physical therapy management of performance-related musculoskeletal disorders in musicians

**DOI:** 10.3389/fpsyg.2014.00706

**Published:** 2014-07-08

**Authors:** Cliffton Chan, Bronwen Ackermann

**Affiliations:** Discipline of Biomedical Science, Sydney Medical School, The University of SydneySydney, NSW, Australia

**Keywords:** physical therapy, playing-related musculoskeletal disorders, work-related musculoskeletal disorders, overuse, performing arts medicine, injury management, formative evaluation, process evaluation

## Abstract

Playing a musical instrument at an elite level is a highly complex motor skill. The regular daily training loads resulting from practice, rehearsals and performances place great demands on the neuromusculoskeletal systems of the body. As a consequence, performance-related musculoskeletal disorders (PRMDs) are globally recognized as common phenomena amongst professional orchestral musicians. These disorders create a significant financial burden to individuals and orchestras as well as lead to serious consequences to the musicians’ performance and ultimately their career. Physical therapists are experts in treating musculoskeletal injuries and are ideally placed to apply their skills to manage PRMDs in this hyper-functioning population, but there is little available evidence to guide specific injury management approaches. An Australia-wide survey of professional orchestral musicians revealed that the musicians attributed excessively high or sudden increase in playing-load as major contributors to their PRMDs. Therefore, facilitating musicians to better manage these loads should be a cornerstone of physical therapy management. The *Sound Practice* orchestral musicians work health and safety project used formative and process evaluation approaches to develop evidence-informed and clinically applicable physical therapy interventions, ultimately resulting in favorable outcomes. After these methodologies were employed, the intervention studies were conducted with a national cohort of professional musicians including: health education, onsite injury management, cross-training exercise regimes, performance postural analysis, and music performance biomechanics feedback. The outcomes of all these interventions will be discussed alongside a focussed review on the existing literature of these management strategies. Finally, a framework for best-practice physical therapy management of PRMDs in musicians will be provided.

## INTRODUCTION

Performing music at an elite level requires greatly developed and integrated sensorimotor and neuromuscular body systems. These are honed by long daily hours of practicing highly complex movements over many years of intensive training (Watson, 2006; Hyde et al., 2009; Krause et al., 2010; Hoenig et al., 2011). As the musician progresses in their skills, the repertoire becomes increasingly challenging requiring more practice time. To then reach the epiphany of the music profession, these meticulous and artistic athletes persist with practice and work at a heightened level of physical stress, making them highly susceptible to neuromusculoskeletal injuries (Brandfonbrener, 2010).

Injury rates are globally reported to be high in this profession (Middlestadt and Fishbein, 1989; Leaver et al., 2011; Paarup et al., 2011; Ackermann et al., 2012), and are thought to relate to the repetitive physical loads reflecting the particular demands of the instrument played. For example, shoulder injuries occur frequently in violin and viola players (Leaver et al., 2011; Paarup et al., 2011; Ackermann et al., 2012), while orofacial and embouchure problems are common in brass players (Iltis and Givens, 2005; Fletcher, 2008; Frucht, 2009). The same phenomenon is observed in sport, whereby the injury region reflects the physical demands of the sport played. For example, shoulder problems are extremely common in swimmers, and knee and ankle problems frequently occur in soccer players (Pink and Tibone, 2000; Drawer and Fuller, 2001; Waivenhaus et al., 2012). Despite this similarity between the task-specific type of injury risks faced by musicians and athletes, there are clear differences in the provision of health education and injury management services.

Sports Medicine has made substantial progress in the monitoring of their athletes for the vigorous demands of their sport and early injury management through strong networks with sports medical and allied health professionals. In contrast, musicians usually have minimal connections with specialized performing arts healthcare professionals (Tubiana, 2000; Guptill, 2011). Throughout their training and professional life, musicians do not receive specialized health education and advice to aid in injury recovery or to minimize potential injury risks (Hoppmann and Patrone, 1989). Furthermore, musicians typically do not participate in other supplementary training activities to support their performance like the athletes. Upon reaching a professional level of performance, musicians usually cease to attend lessons or receive any technical feedback or instruction. This is unlike other elite performance domains, such as sports and dance, where this is maintained throughout their career. Increasing knowledge of healthy practice strategies and implementing tailored injury prevention measures specifically for musicians, following approaches similar to Sports Medicine, may reduce the susceptibility of musicians to a range of musculoskeletal disorders (Zaza, 1993, 1994; Tubiana, 2000).

In addition to exposure to physical injury risks, musicians, like other athletes, face intense psychological pressures arising from performing under public scrutiny. Unlike sports communities, however, negative cultural perceptions regarding injury within the musical community can provide a challenge to implementing a best practice model of healthcare (Rickert et al., 2013). Negative connotations of inferior technical competency are commonly directed at musicians suffering performance-related injuries by their peers (Guptill, 2011, 2012; Raymond et al., 2012). Injuries can thus be associated with feelings of professional inadequacy or shame (Bragge et al., 2006; Guptill, 2011; Chimenti et al., 2013), tending to lead to injury concealment behaviors that delay the implementation of appropriate healthcare management. Such stigmas associated with playing-related injuries, as well as the lack of specialized health services and education, have led to professional orchestral musicians’ often playing through the pain until they can no longer manage (Schoeb and Zosso, 2012). Additionally, there is increasing awareness of the complex relationship between performance-related musculoskeletal disorders (PRMDs), performance-related pain and a range of psychosocial factors (Altenmüller and Jabusch, 2010; Kenny and Ackermann, 2013). Hence effective rehabilitation of musicians’ injuries requires healthcare professionals to manage both the physical and psychological aspects of PRMDs (see **Table 1**). Healthcare practitioners should be aware of their potential contribution to psychosocial and cultural factors in the performing arts field, however, it is beyond the scope of this paper to explore the psychosocial interventions for PRMDs.

**Table 1:** Physical and psychosocial factors influencing the development and perpetuation of performance-related musculoskeletal disorders (based on Wu, 2007; Altenmüller and Jabusch, 2010; Brandfonbrener, 2010; Leaver et al., 2011; Ackermann et al., 2012; Kenny and Ackermann, 2013).

Physical risk factors		Psychosocial risk factors
Non-modifiable	Minimally modifiable or modifiable	
• Instrument played	• Overload – sustained high levels of playing or sudden increases in playing load	• General and/or performance anxiety
• Anthropometrics		• Depression
• Gender		• Pressures from self, peers, educational institution or work organization
• Playing conditions – temperature, length of rehearsals and performances	• Lack of rest breaks in rehearsals and private practice	• Work and/or non-work Related stress
• Joint laxity - past trauma or generalized	• Poor posture	• Social phobia
• Challenging repertoire	• Poor biomechanics	• Personality traits – e.g., somatization tendencies, extreme perfectionism
	• Joint hypomobility	
	• Instrumental technique and pedagogical style	
	• Lack of physical conditioning	
	• Poor injury management	

Physical therapists are trained to use scientific and evidence-based assessments and treatments to prevent musculoskeletal disease and disability, and are experts in optimizing function and performance (Kolt and Snyder-Mackler, 2007). As a profession trained to treat a wide range of neurological, respiratory and musculoskeletal conditions, they are well-positioned to apply their manual therapy, exercise and biomechanics skills to managing PRMDs in musicians (World Confederation for Physical Therapy, 2011). However, the pool of physical therapists treating musicians as a specialty is very small compared to sports physical therapy although this specialty appears to be growing. One of the challenges to current physical therapy management approaches is the lack of specialized training programs and available evidence on effective intervention strategies for musicians.

## USE OF FORMATIVE AND PROCESS EVALUATION METHODOLOGY

The use of rigorous epidemiological approaches in the design and implementation stages of developing an occupation-specific prevention and management program may increase the likelihood of intervention outcomes being successful (Viera and Kumar, 2004; Stetler et al., 2006; Boocock et al., 2007; Jurg et al., 2008; Bell and Burnett, 2009; Harding et al., 2009). This was found to be the case in our studies with professional orchestral musicians (Chan et al., 2013b,c). All stakeholders including researchers, expert clinicians, orchestral management and musician employee representatives should contribute. Including all relevant parties in the development of prevention and management programs can help to avoid organizational or work barriers that may otherwise hinder trial compliance and participation rates (Dehar et al., 1993). Formative and process evaluation methodologies are increasingly being employed to ensure interventions are researched, designed and pilot tested prior to implementation (Stetler et al., 2006; Jurg et al., 2008; Baranowski et al., 2009), and suit the needs of designing interventions for the musician population.

In the first stage, formative evaluation strategies were utilized in the development of specific interventions for professional orchestral musicians to optimize likely positive outcomes (Chan et al., 2013b,c). A comprehensive literature review of musicians’ health, as well as a review of relevant literature from occupational and sports medicine was undertaken and information synthesized to draft a preliminary program. A panel of experienced physical therapists reviewed the proposed intervention programs and were asked for their evaluation and comments. Musicians and their management teams were also asked to give feedback about the proposal. In instances where there was a lack of consensus, the program was revised, and subsequently sent out for feedback. Working closely with orchestra management staff was necessary for the programs to be designed in a way that was compatible with the context and practicalities of the orchestral environment and schedules. This formative evaluation process was vital in ensuring the interventions were highly credible and likely to optimize desired results.

In the pilot testing or process evaluation phase, interventions were trialed and modified as necessary based on evaluation of the intervention by the researchers, feedback from the physical therapists involved in the study as well as feedback from a small number of musicians that had volunteered for the pilot trial. While these physical therapists were highly experienced and qualified in sports and occupational physical therapy, most did not have musician-specific injury management expertise. This stage of the program development had the additional benefit of allowing participating physical therapists to familiarize themselves with the research protocol and intervention delivery. The piloting process was also useful in refining the intricacies of implementing the intervention and identifying potential impracticalities or errors that may occur when implementing the program on a larger nationwide scale. Therefore, formative and process evaluation provided an effective means to develop intervention trials that had good support from all relevant stakeholders at the outset of the project.

The purpose of this review is to inform physical therapists of evidence-based management strategies for PRMDs in the musician population that can be readily implemented in the clinic environment and introduced into music institutions and organizations. The outcomes of a series of formative and process evaluated intervention strategies, undertaken as part of the “Sound Practice” project, along with their clinical and research implications will be presented. These include: health education and advice provided to musicians, specialized onsite injury and recovery services, exercise regimes, and postural and biomechanics analysis.

## RESULTS

### MUSICIAN’S HEALTH EDUCATION AND ADVICE

It is generally accepted that a vital component in the prevention and management of work-related musculoskeletal disorders is appropriate health education and medical advice (Bohr, 2002; Silverstein and Clark, 2004). Since professional musicians often endure long rehearsals and performances that involve extremely repetitive activities, it is only sensible to educate them about potential risks to which they may be exposed; since these could lead to them sustaining a work-related injury (Pascarelli and Hsu, 2001; Baldwin, 2004; da Costa and Viera, 2010). For example, some modifiable risk factors associated with PRMDs on which physical therapists could provide advice are: scheduling of private practice sessions, rest and relative rest after injury, basic nutrition and hydration, general fitness and early injury identification and management. However, at present there is a lack of formal health and fitness education during musical training as well as within the orchestral workplace (Barrowcliffe, 1999; Tubiana, 2000; Dommerholt, 2009). Musicians’ lack of understanding of injury causes or best management approaches may lead to musicians using unreliable sources for health advice, and therefore poor or inadequate management of injuries. Physical therapists working with professional musicians should be able to provide specialized and relevant healthcare advice to promote optimal injury prevention strategies and management enabling musicians to safely sustain their necessarily highly repetitive playing loads.

Throughout the duration of the “Sound Practice” project each of the Australian state orchestras received regular delivery of healthcare education, covering physical, psychological, nutritional and auditory health topics. Informal feedback following these sessions indicated the musicians felt they had a high need for this education, and should have received it much earlier in their musical careers.

#### Private practice scheduling

The importance of planning out private practice for the prevention of PRMDs is suggested by various authors (Zaza, 1994; Green et al., 2000). Even professional musicians may not consider that challenging and higher intensity repertoire should be practiced in shorter durations with frequent rest breaks or with easier repertoire in between (Fry, 2000) to avoid muscle fatigue. While professional orchestral musicians often have limited flexibility in their private practice schedule, wherever possible, distributing the total private practice schedule through the day (Green et al., 2000) will ensure that there is adequate rest and recovery for the body, and allows for better skill refinement and consolidation (Donovan and Radosevich, 1999; Robertson et al., 2004; Lee and Wishart, 2005).

When the amount of playing hours fluctuate due to performances, auditions and other playing demands, musicians should be made aware of the potential overload on musculoskeletal structures. Such a problem is often caused by sudden increases of overall playing load and musicians should adjust their practice schedules accordingly (Newmark and Lederman, 1987; Davies and Mangion, 2002; Ackermann and Adams, 2004). During periods of increased load, a higher number of performances and rehearsals, musicians may need to reduce physical practice and employ the use of practice strategies such as shadow-playing or mental practice (Menuhim, 1986; Keller, 2012). In the opposite situation, after periods of minimal playing (i.e., during holidays), musicians should build up their intensity and duration of private practice prior to returning to full playing workloads (Green et al., 2000). In summary, orchestral musicians should carefully plan their private practice schedules as well as monitor their overall playing load to minimize the potential for development of PRMDs.

#### Rest and relative rest after injury

Rest breaks to prevent work-related musculoskeletal disorders, especially injuries relating to overuse as is typical in professional musicians, are important in all occupations involving long periods of repetitive work (Huang and Feuerstein, 2004). In occupational medicine literature, it is recommended that regular breaks be taken, a minimum of 5 min every hour, to prevent excessive physical stress and allow energy stores in the muscles to be replenished (Westgaard and Winkel, 1996; Silverstein and Clark, 2004; Kennedy et al., 2010). For musicians, frequent and regular breaks assist in reducing the constant strain and load-bearing on the joints, as well as allowing recovery of supporting musculature and fine-control muscles of the fingers and lips (Zaza, 1994). While musicians may have little control over rest breaks during orchestral rehearsal and performance, they should be able to appropriately implement these in their private practice sessions.

There is evidence to suggest that taking regular breaks during private practice has a protective effect on recurrent PRMDs in musicians (Zaza and Farewell, 1997). As a general recommendation, a 5-min rest break should be taken for every 25 min of playing (Zaza, 1994; Robinson and Zander, 2002; Ackermann, 2010). Musicians should also take into consideration more frequent breaks when practicing repertoire with higher intensity and increased difficulty. If practice sessions are longer, 10–15 min rest should be taken after 45–60 min (Robinson and Zander, 2002; Ackermann, 2010). Musicians should be acutely aware that working at sustained elevated physical-stress levels is damaging to musculoskeletal structures and without adequate rest the tissue breakdown process will exceed the speed of repair ultimately leading to injury (Kumar, 2001).

Following an injury, it is important that the musician understand the basic healing characteristics of the relevant body tissues to ensure good compliance in rehabilitation protocols (Ackermann, 2010; Hoppmann, 2010). In the event of acute musculoskeletal injury, it is recommended that tissues should be given a rest period of between 3 and 7 days to optimize the initial inflammatory phase (Kannus et al., 2003; Järvinen et al., 2007). To facilitate optimal tissue healing and integrity and prevent further tissue atrophy the rest period should be followed by gentle and graduated range of movement exercises depending on the severity of tissue damage (Popovich et al., 2000; Orchard and Best, 2002; Kannus et al., 2003; Nash et al., 2004). For the injured musician, shorter practice sessions with more regular breaks may be necessary in the early injury recovery phase (Norris, 1993), for example 5 min playing followed by 5 min rest. As symptoms subside and the injury heals, the number of playing sessions and their duration can be progressively increased to match ability. Rehabilitation should be directed toward functional recovery from the outset as in other specialized domains, aiming for graduated return to work from about week six following the injury, depending on the injury severity (Järvinen et al., 2007). Orchestral musicians may otherwise return to performance prior to adequate healing and thereby risk ongoing health issues.

#### Nutrition and hydration

It would seem logical that nutrition and hydration are important considerations in a musicians’ preparation before their long practice sessions and performances much like the athletic population. A musician’s nutritional needs are likely to be above that of the general population due to the physical nature of their work over long periods suggesting nutritional education should be included in the prevention and management of PRMDs (Robinson and Zander, 2002; Shafer-Crane, 2006).

In low intensity endurance activities, approximately 60% of the energy expended comes from carbohydrate sources (Holt, 1993; Manore et al., 2009). This would suggest that before rehearsals and performances, a musician’s diet should include carbohydrates, then fats and proteins. Low to medium glycemic index (GI) carbohydrates, i.e., energy sources that produce a slow to moderate rise in blood glucose and insulin, would be likely to be the most ideal to enable the energy to be sustained over long rehearsals and performances (Manore et al., 2009). Fat and protein sources provide approximately 25 and 15% respectively of the energy supplies during low intensity endurance activity (Holt, 1993; Manore et al., 2009). Following long performances, consuming a more rapid release carbohydrate food source (medium to high GI) as well as proteins are suitable to optimally replenish depleted fuel reserves and to facilitate repair of any muscle fiber breakdown that may have occurred (Phillips, 2006; Campbell et al., 2007; Kumar et al., 2009; Manore et al., 2009). Sports science research indicates it is important to consume these carbohydrate and protein food sources within one hour after the activity (Campbell et al., 2007). Therefore, an adequate nutritional intake before and after strenuous rehearsals and performances may be important in potentially reducing the risk of PRMDs (Phillips, 2006; Campbell et al., 2007).

Another important component of nutritional consideration is water intake, with these needs varying from approximately 2 l of water per day for a sedentary adult male under normal environmental conditions up to approximately 3 l with the addition of modest physical activity (Kenefick and Sawka, 2007; Jéquier and Constant, 2010; Popkin et al., 2010). To maintain hydration in a warmer environment, water intake may need to be further increased to account for the greater fluid loss through sweat. If musicians become too dehydrated prior to performances, this could lead to tiredness, muscular weakness, dry and sticky mouth and tongue, headaches, dizziness or light-headedness (Jéquier and Constant, 2010), which could potentially affect playing. Musicians who regularly perform under different environmental conditions both indoors and outdoors should be mindful of their water intake; replenishing before, during and after playing as required (Kenefick and Sawka, 2007; Montain, 2008). Even a small degree of dehydration can affect cognitive and physical function (Shirreffs, 2009; Popkin et al., 2010), and as such musicians needing to perform at their peak and reduce the likelihood of injury should be aware of their water intake.

#### General fitness

Participation in cardiovascular fitness and resistance training has been suggested to be an important element in maintaining a healthy and long career in the performing arts (Shafer-Crane, 2006). There are many physical and psychological benefits associated with appropriate levels of regular physical activity, such as significant increases in cardiovascular fitness, skeletal muscle endurance, reaction time, and decreased incidence of osteoarthritis, depression and anxiety (Booth et al., 2012). In one previous survey, musicians who performed physical activity regularly rated their perceived exertion level during rehearsal to be significantly lower than musicians that did little or no physical activity (Wilke et al., 2011).

Musicians, like other hyper-functioning performers such as dancers and athletes, should undertake both cardiovascular and resistance exercises each week to best achieve and maintain optimal physical conditioning. Based on recommendations from the American College of Sports Medicine (Medicine ACoS, 2010) and expert music health practitioners (Ackermann et al., 2002; Ackermann, 2010; Wilke et al., 2011), an example of a potential best-practice exercise guideline for musicians is included in **Table 2** below including the type, frequency, and duration of exercises.

**Table 2:** An exercise guideline for musicians to improve cardiovascular fitness and muscular conditioning.

Type of exercise	Frequency and duration	Example exercises
Cardiovascular (aerobic) fitness exercise	Five sessions of moderate intensity exercise per week, at least 30 min per session or three sessions of high intensity exercise per week, at least 20 min per session	Brisk walking, cycling at an easy pace, swimming leisurely, or jogging, cycling with a slight incline or low resistance, swimming with a moderate effort
Resistive (muscular endurance) exercise	Two sessions per week, 2–3 sets of 10–20 repetitions, with 90 s rests in between sets Aim to target 8–10 major muscle groups each week	• Scapular retractors (seated rows, reverse flyes)
		• Shoulder external rotators
		• Low back extensions
		• Hip extensions (bridging in supine lying)
		• Leg press, squats, or lunges
		• Tricep extensions
		• Bicep curls, push ups, chest press^*^

* Should be performed less regularly as the muscles used in these exercises are commonly tight and overused from instrumental playing.

Specialized and tailored musician exercise programs may further enhance physical condition of performers without overloading already heavily worked structures, and these will be discussed in Section “Cross-Training Exercise Regimes” below.

#### Early injury identification and management

Early identification of injury and commencement of rehabilitation is key to optimizing prognosis of most neuromusculoskeletal problems (Linton, 2002; Gatchel et al., 2003; Stucki et al., 2005), with potential benefits to professional orchestral musicians (Milanese, 2000). Not only can the best healthcare management be immediately implemented when injury presents (Orchard and Best, 2002; Dommerholt, 2010; Pemoff et al., 2012), but secondary problems can also be prevented (Laisné et al., 2012). For musicians, early injury identification or triage by an onsite physical therapist could minimize the effect of the injury on playing/performance by immediately implementing a plan for best injury management (Chan et al., 2013c), thus aiding a more rapid return to work or play (Shafer-Crane, 2006). Musicians should be educated on the principles of first aid that can be applied prior to a healthcare consultation for acute injury management. The principles of first aid could include: resting the injured area, icing the injured area, and applying compression with elevation in the presence of swelling and seeking a diagnosis if symptoms persist (Australia SJsA, 2012; Bruckner and Khan, 2012).

For musicians, receiving immediate and specific advice when to simply rest and self-manage a mild strain or when to consult a health professional for an injury is likely to be important for optimizing recovery.

### SPECIALIZED ONSITE INJURY AND RECOVERY SERVICES

A brief intensive physical therapy-led triage service for professional orchestral musicians was successful in managing musicians’ injuries during a busy playing period (Milanese, 2000). This author concluded that the availability of a regular triage service may allow earlier identification and management of PRMDs occurring throughout the usual playing schedule. Such an acute injury management advice service, led by physical therapists, was developed and implemented for 12-weeks for each state orchestra Australia-wide (Chan et al., 2013c). In consultation with orchestral management, clinics were held for one hour every fortnight, usually during the lunch hour between rehearsal calls, with both an anonymous appointment booking system or drop-in services available.

This onsite triage service was well-received by the musicians, and was evidenced by consistent feedback of the musicians’ gratitude toward having such an easily available injury management service. Most of the consultations at these triage services were classified as PRMDs, and services were more likely to be utilized by females and string players. Most musicians who presented with PRMDs reported that these affect their normal playing, and the physical therapists considered that the majority of conditions seen may have been preventable. These encouraging results support the regular accessibility of a triage clinic at orchestral premises; if this is achievable in the longer term it may be possible that many PRMDs could be better managed or prevented altogether.

Following on from the injury advisory service, an intensive trial providing both injury advice and short treatments (usually recovery massage) were undertaken with one orchestra. This orchestra’s management team forecasted a possible increase in playing-related injury due to a heavier than normal orchestral cycle immediately following a holiday period. In this study of short recovery treatments and injury advice, 10 to 15 min consultations with a qualified massage therapist and/or physical therapist were made available throughout the duration of the orchestral season. Feedback from musicians and orchestra management indicated that most musicians benefited from these sessions. Additionally, shorter sessions of lighter “eﬄeurage and petrissage” style massage (Weerapong and Kolt, 2005) were preferred over the occasional more intensive massage or treatment approaches as the latter tended to leave some soreness effects in the next playing session. Management reported far less absences than they had anticipated over the course of the trial. These quick recovery treatments may be greatly beneficial to musicians during busy playing periods, or during intensive touring programs (Ackermann, 2002).

### CROSS-TRAINING EXERCISE REGIMES

Despite suggestions that purpose-designed exercise regimes may play a major preventative role in avoiding PRMDs (Zaza, 1994; Brandfonbrener, 1997; Foxman and Burgel, 2006), there is a lack of clinical trials in this area for professional orchestral musicians. In other occupational health literature, there is a strong body of literature indicating the efficacy of exercise therapy in targeting work-related musculoskeletal disorders, especially in the neck, lower back and upper body regions (Boocock et al., 2007; Blangsted et al., 2008; Gerg and Smith, 2008; Äng et al., 2009; Bell and Burnett, 2009; Machotka et al., 2009; Andersen et al., 2010a,b; Lysaght et al., 2010). Purpose-designed exercise programs appear to be equally effective or better at reducing pain and improving functional outcomes in a cost-effective manner than a wide range of manual therapy or ergonomic interventions (Verhagen et al., 2007; van Eijsden et al., 2009). For these reasons, it is worth thoroughly investigating the efficacy of participation in tailored exercise programs for the professional orchestral musician population.

To guide the development of a specific exercise program for professional orchestral musicians a review of the existing literature was performed. Only one intervention trial was found that investigated the effects of a 15-week exercise program on PRMD levels and associated risk factors in a small sample of 17 professional orchestral musicians (de Greef et al., 2003). The intervention did not specifically evaluate exercise, but it was part of an intervention package of musician-specific education, some specific exercises and a traditional general strengthening exercise program. These authors found significant reductions in PRMD levels and self-reported improvements in playing-related posture, strength, fatigue, anxiety and ability to cope with work-related stress. Three other studies investigated the effect of exercise programs on university music students. Exercise interventions included resistance, core stability or aerobic exercises and were reported to reduce the presence, frequency and intensity of PRMDs and to improve instrumental playing posture (Spahn et al., 2001; Ackermann et al., 2002; Kava et al., 2010). It appears that simply participating in generic aerobic and strengthening fitness programs that provide overall cardiovascular and strength benefits are insufficient to prevent PRMDs or improve instrumental performance in musicians (Van Hees, 1997; Zetterberg et al., 1998). These findings from the four exercise trials suggest that there is a key role for developing exercise programs to target and potentially prevent PRMDs in professional musicians (Andersen et al., 2008).

The intervention groups participating in an exercise program targeting specific body regions susceptible to injury in musicians reported positive benefits in relation to PRMDs, exertion and a range of playing-related factors (Chan et al., 2013b). The literature on common body regions of PRMDs experienced by musicians was used to identify target regions for strengthening (Roset-Llobet et al., 2000; Wu, 2007; Leaver et al., 2011; Paarup et al., 2011; Ackermann et al., 2012). Existing evidence from sports and occupational health literature and well-accepted clinical practice was integrated to produce a progressive exercise program aimed to increase the endurance of supportive musculature in the shoulder, neck, abdominal, lower back, and lumbo-pelvic regions (Chan et al., 2013b). In addition, the exercises incorporated improving motor control and movement patterning of the body region. Participants were taught how to activate weakened stability muscles and then incorporate these muscles in a task-specific and functional manner for musicians.

Exercise programs targeted toward strengthening the supportive musculature of commonly injured areas in musicians were effective in reducing PRMDs and improved numerous other playing-related factors. The evaluation of survey results from the DVD exercise trial was greatly encouraging. A statistically significant reduction in PRMD frequency and severity occurred immediately after the 12-week intervention (Chan et al., 2014a). In the face-to-face exercise intervention study similar positive benefits were seen. Additionally, the opposite was observed in the control group’s PRMD frequency and severity scores, which increased over the time of two standard orchestral cycles despite most of them undertaking regular physical activity (Chan et al., 2014b). After the cessation of the intervention at six-month follow-up, the beneficial effect seen in the intervention group slowly declined. This slight decline in positive effects of exercise is consistent with other literature documenting that exercise levels must be maintained for ongoing benefits (Kay et al., 2005; Fransen and McConnnell, 2008; Macedo et al., 2009; Vina et al., 2012). Participants of the interventions also reported moderate to high perceived improvements in the strength of the muscles that support their playing, flexibility, posture and ease of movement after the interventions, with most of these benefits maintained at similar levels at follow-up. The program appeared to be safe, with no injuries incurred as a direct consequence of the exercises. Therefore, it appears that musicians benefit from undertaking targeted exercises for muscles that support their instrumental playing demands and then to maintain such a program to prevent or manage PRMDs.

The ability of musicians to consistently participate in exercise programs was identified as a challenge by both orchestral management and musicians due to the continued variability in their work schedule. Programming of such sessions was an important and often complicated component that has to be factored into the successful implementation of such an intervention. By undertaking face-to-face exercise classes immediately before or after orchestral rehearsals and ensuring exercises focused on supporting rather than playing musculature, participants were still able to begin their rehearsal without any complaints of muscular fatigue affecting performance (Chan et al., 2014a). An additional solution suggested in the sports and rehabilitation medicine domains was to deliver an exercise program via the use of digital media (Hupperets et al., 2009; Vandelanotte and Mummery, 2011; Khalil et al., 2012). Such a flexible delivery method may increase accessibility, and allow more self-conscious musicians to participate without the scrutiny of their peers. Consequently, with the assistance of a professional film crew, the authors (CC and BA) produced an exercise DVD based on the program outlined in Chan et al. (2013b). The DVD study had a much higher initial uptake across all orchestras, although tended to be more popular with the musicians who had an existing physical activity regime (Chan et al., 2014a), suggesting that face-to-face classes are still also useful for musicians who perhaps feel they need more guidance and supervision. Through careful scheduling of the exercise class around rehearsal times and different forms of intervention delivery modes, barriers to attendance could be overcome.

### PERFORMANCE POSTURE ANALYSIS

Correct posture has been defined as the body position adopted that loads the joints safely, helps conserve energy and allows freedom of movement (Kendall et al., 1952). Throughout instrumental playing, such a correct posture needs to also allow supportive muscles to sustain efficient static or dynamic movements and stability of the joints during performance actions. However, achieving optimal posture while performing can challenge basic concepts of “ideal” posture as playing most musical instruments requires maintaining asymmetrical postures in either sitting or standing over prolonged periods of time (Cailliet, 1990; Haslegrave, 1994; Nyman et al., 2007; Claus et al., 2009; Edling and Fjellman-Wiklund, 2009; O’Sullivan et al., 2012). Non-ideal postures requiring higher levels of muscle activation to support the musician and their instrument while compensating for reduced balance and control, may increase static loading and stress of neuromusculoskeletal structures, leading to earlier muscular fatigue and excessive muscular tension, creating a higher risk of developing PRMDs (Magnusson and Pope, 1998; Medoff, 1999; Kapandji, 2000; Tubiana, 2000; Kumar, 2001; Quarrier and Stenback, 2002; Price and Watson, 2011).

When maintaining postures for extended periods of time, such as sitting which is common to all orchestral musicians, spinal structures may be in non-ideal positions and muscles may become overloaded (Hedman and Fernie, 1997; Briggs et al., 2007). The effect of these postures on loading is further compounded by the added dynamic and asymmetrical stressors of playing their instrument (Hides, 2004; Briggs et al., 2007). The accumulation of these factors may accelerate degenerative processes in spinal motion segments and contribute to the development of dysfunction and pain (Kendall et al., 1952; Eijsden-Besseling et al., 1993; Hoogendoorn et al., 2000; Briggs et al., 2007; Price and Watson, 2011). Hence, maintaining as neutral and yet supported posture as possible may be important to prevent PRMDs in professional orchestral musicians.

Not only are sustained and poor postures a potential cause of injury to musculoskeletal structures, such postures could also potentially affect the neuromuscular system leading to an inferior musical performance. Posture also plays a crucial part in breathing mechanics, suggested to influence the volume and quality of sound produced by woodwind and brass instrumentalists (Quarrier and Stenback, 2002; Gaunt, 2004; Ackermann, 2010). In the general population, slouched posture has been shown to increase respiratory effort, and significantly decrease breathing capacity and control (O’Sullivan et al., 2002; Landers et al., 2003), with likely altered abdominal and accessory respiratory muscle recruitment patterns, as well as non-optimal biomechanical positioning of these muscles (Roussos, 1985; Kera and Maruyama, 2005; Ainscough-Potts et al., 2006; Ratnovsky et al., 2008). Such changes may have a negative impact on breathing endurance and control for musicians, although more research is needed in this area.

In order to test the impact of postural changes on instrumental playing, it was important to first ascertain whether clinically utilized methods of assessment were a reliable way of measuring and monitoring postural change. A postural trial was undertaken to determine whether experienced observers, including healthcare professionals and music educators with Alexander Technique/Body Mapping training, were able to detect postural changes in musicians from photographs following a 10-week exercise intervention program. This qualitative approach was tested because health professionals commonly use anterior and lateral photographic views of posture as part of their re-assessment of intervention effectiveness. Our results suggest that both health professionals and music educators were statistically significantly better (*p* < 0.001 and *p* = 0.002 respectively) than chance at selecting the true post-intervention photograph (Chan et al., 2013a). Although the health professionals (66%) were slightly better than the music educators (60%) at selecting the true post-intervention as having better posture, this result was not statistically different. Interestingly, 97% of the musician participants (*n* = 57) in this trial reported a noticeable improvement in their playing-related posture, which may reflect the limited ability for static photographs to evaluate dynamic postural changes and underlying muscular tension changes perceived by the professional orchestral musicians. While the use of static photographs may be useful in observing large scale abnormal skeletal postures, such as spinal deviations, forward head postures and shoulder angles (O’Sullivan et al., 2012), more subtle changes in a musician’s technique and posture may suit other assessment methods such as videography. Nonetheless, the use of photographs may still provide a useful component of the re-assessment of a musician’s posture by experienced health practitioners and music educators.

### MUSIC PERFORMANCE BIOMECHANICS FEEDBACK

The term “biomechanics” refers to the study of internal and external forces acting on the human body and the effects produced by these forces (Freivalds, 2011). In occupational biomechanics, the physiological loads and stresses placed on the human body as a result of work-related tasks are analyzed (Chaffin et al., 1991). This can provide useful insights for physical therapists to help musicians optimize playing-related posture, maximize playing technique efficiencies, understand the importance of ergonomics and use of equipment, and potentially minimize unnecessary load on body preventing injury (McGinnis, 2013). Biomechanical analysis has also been applied extensively to sports performance, and in collaboration with coaches has led to new advances and insights into performance optimization and injury prevention. The term “Music Performance Biomechanics” is used here to extend this concept to the study of the mechanics of human movement applied to musical performance.

Whilst laboratory based quantitative research on music performance biomechanics is continuously emerging (Visentin et al., 2008; Chadefaux et al., 2012; Fernandes and de Barros, 2012; Kelleher et al., 2013; Sung et al., 2013), music teachers still rely almost entirely on qualitative approaches to performance technique feedback. This is mostly based on the outcome goal of the quality of sound output rather than observing how the musician is moving and reacting to achieve the desired sound. Even with the far greater amount of technological data regarding the athletic performer, there is little evidence as yet on how to best utilize such information to provide effective feedback in performance situations (Phillips et al., 2013). A qualitative approach is typically used in the practical sport setting, whereby movements and elements of technique are analyzed and then remediation occurs through feedback to address any identified flaws that may detrimentally affect performance or increase the risk of injury (Lees, 2002). Experts consider that observing the movements of the performer with their instrument is a key part of health assessment of the musician (Blum, 2003). Despite this, developing reliable rating scales for observing musicians playing their instrument has provided only limited success, with complicated systems showing poor inter-rater reliability (Ackermann and Adams, 2004), and simpler analyses showing better reliability (Driscoll and Ackermann, 2012). In such a domain where sound quality is the primary goal and playing styles can be diverse, it seems sensible to take a collaborative approach incorporating the expertise of the musician and the healthcare professional in achieving the goal of improving both sound and movement quality during performance.

The aim of the biomechanics feedback study as part of the “Sound Practice” project was to videograph performers in their usual rehearsal situations, identify those actions that may create a higher risk of physical strain within an individual musicians’ technique, and then discuss the footage and analysis with the performer. The approach of providing individualized biomechanics feedback aimed to incorporate strategies is suggested by current research, whereby feedback needs to be task and person specific and the key elements that may benefit from remediation being readily modifiable (Phillips et al., 2013). The use of observation offers a chance to process performance information that could not occur simultaneously with physical practice, facilitating refinement (or maintenance) of complex motor skills (Wulf et al., 2010). In sports medicine literature, immediate feedback has been shown to enhance athletic task performance (Argus et al., 2011). By providing immediate feedback of the musician’s performing as well as using the experience and skills of the musician themselves in the process, may then further improve the ability to adapt recommendations into an individual technique and optimizing musical performance.

This biomechanics feedback procedure was further refined using process evaluation by piloting methods of feedback with an orchestra not involved in the “Sound Practice” project. Semi-structured interviews post-feedback were used to further refine the amount of feedback given and presentation of footage and other information. 60 musicians volunteered to participate in this trial from the five orchestras who responded to the research invitation. Four out of these five orchestras requested a repeat of the trial in response to positive feedback from the musicians. A simple post-feedback survey was used to evaluate the trial, ranging from -5 for negative impact to +5 for positive impact. Of the 50% of musicians who responded to the survey, every response was neutral or positive in terms of impact on performance. The self-report factors included: ease of playing; effect on pain; muscle tension; understanding playing actions; impact on playing posture; impact on muscle fatigue; and impact on playing biomechanics. A narrative section allowed musicians’ to write any additional responses, and these reinforced the positive outcome of this intervention and the strong engagement by the musicians in the process. Overall, this final biomechanics feedback trial was welcomed and supported by the orchestra management and the musicians themselves, and appeared to be a proactive way of monitoring performance and preventing injury.

While more research is needed in this field, along with exploring methods of integrating qualitative and quantitative data, preliminary results are encouraging and reinforce the need for physical therapists to consider music performance biomechanics as a component of their health management of the musician.

## RECOMMENDATIONS FOR EFFECTIVE PHYSICAL THERAPY MANAGEMENT OF PERFORMANCE-RELATED MUSCULOSKELETAL DISORDERS IN MUSICIANS

Current approaches to the management of PRMDs have been based on expert opinion (Zaza, 1993; Shafer-Crane, 2006; Dommerholt, 2009, 2010) and translational research adapted from other fields of medicine. However, as guidelines for practice emerge based on new research findings (Altenmüller and Jabusch, 2010; Driscoll and Ackermann, 2012) physical therapists should aim to adapt and modify their practice using such guidelines to enhance their management of PRMDs in musicians.

At all stages of physical therapy interventions for musicians’ injuries, from prevention to assessment and onto rehabilitation, the performer must be regarded as a highly trained and skilled individual. Our own approaches to management need to be expanded and specialized to better meet the needs of the musician population. During history taking, the musician’s background of years of playing, stage of skill, practice habits, teacher and “school of playing” should be recorded as these may influence their playing technique and posture. This information can add valuable perspectives for why the musician is adapting postures that may place them at a biomechanical disadvantage or provide indications to the mechanism of injury. During the physical examination, musicians must be examined with their instrument and with attention to the extreme ranges of motion related to performance requirements (Driscoll and Ackermann, 2012). A summary of some of these important information specifics to assessment and treatment of musicians is illustrated in **Table 3**.

**Table 3:** Management of the injured musician.

Assessment	Treatment
**History**	**Education and advice**
• Years of playing on primary instrument	• Private practice scheduling
• Stage of skill on primary instrument	• Rest and relative rest after injury
• Increased switches between instruments or recent change of primary instrument	• Nutrition and hydration
• Current and past teacher/s	• General fitness
• “School of playing”	**Specialized onsite injury and recovery services**
• Total playing hours (the sum of private practice, rehearsals and performances)	• Music organizations and music educational institutions should consider implementing such a service to ensure musicians received specialized advice on the best course of action for any concerns and injuries, as well as immediate management by suitably experienced healthcare professionals.
• Preparation routine	
• Practice schedule (e.g., 1 h, twice per day) and the duration and frequency of rest breaks	
• Changes to repertoire (e.g., style, difficulty)	**Cross-training exercise regimes**
• Any recent modifications to instrument (e.g., ergonomic devices) or playing technique	• Musicians should undertake a targeted exercise program for any existing postural concerns or identified problems, and for strengthening supportive musculature required for their instrument.
• Impact of pain on current playing capacity	
• Other relevant work-related psychosocial risk factors	**Music performance biomechanics feedback**
**Physical examination**	• This tool could be used as a monitoring system to track progress or provide feedback to the musician, student or teacher.
• Observation of static posture (with and without instrument) and playing posture	
• A “Performance Postural Analysis“ of the musician with and without instrument in sitting and standing should be performed. Poor postural habits are often missed if not performed under playing conditions (e.g., forward head posture when trying to seal their embouchure with the interface of the woodwind/brass instrument). Ideally this should be done with videography but photographs can also be used for more gross postural issues.	**Ergonomic considerations**
	• Sourcing instrument-specific ergonomic modifications to aid the adaptation of the instrument to the musician.
• Measure available range of movement at the injured joint to ensure there is sufficient range for the instrumentalist (e.g., 99. of supination at left elbow in violin players, left hand span larger than right hand span in cello and double bass players).	
• Test muscle strength and control of supporting muscles relevant to their instrumental playing and problem (e.g., string player with shoulder issue – test external rotator cuff versus internal rotator cuff strength)	

Physical therapists should carefully structure the musicians’ rehabilitation program to include musician specific education and advice, targeted exercise regimes and any other intervention strategies to achieve better functional outcomes. After an injury, musicians should be informed of the need to implement a graduated return to playing and/or return-to-work plan such that sufficient rest and recovery for any damaged neuromusculoskeletal structures. A practice diary could be used to record and monitor timing of playing and rest breaks, Additional strengthening, movement or flexibility regimes off the instrument into relevant ranges of motion, and muscle activation patterns specific for the instrumentalist (e.g., consider whether the strengthening needs to occur in inner/middle/outer range for the muscle/s with a which type of muscle contraction). Consideration of playing-related demands created by the instrument and the workplace may require additional management strategies (e.g., reduced rest week between multiple orchestral cycles with increased performances and difficulty of repertoire may require an onsite physical therapy recovery service to aid recovery and promptly manage any playing-related injuries).

In summary, emerging evidence in the field of music medicine has lead to useful insights and clinically applicable strategies for the management of PRMDs in musicians. Beyond a thorough understanding of current models of management of work-related musculoskeletal disorder, physical therapists should further tailor their history taking and physical assessment to treat musicians as a highly specialized and hyper-functioning population. Relevant education and advice should be provided to musicians early in their injury whilst preventative information needs to be delivered early and throughout their careers. Specific training and workplace demands must also be carefully considered in the comprehensive management of the musician’s injuries. Finally, proactive steps at music institutions and organizations should be taken, such as implementing onsite health prevention and management services for musicians, as playing-related problems in this population is not only highly prevalent and persistent but can also become greatly debilitating ultimately jeopardize a musicians’ career.

## AUTHOR CONTRIBUTIONS

Cliffton Chan and Bronwen Ackermann made substantial contributions to the conception and design of this work, drafting the work and revising it critically for important intellectual content. We give permission for this paper to be published and agree to be accountable for all aspects of the work in ensuring that questions related to the accuracy or integrity of any part of the work are appropriately investigated and resolved.

## Conflict of Interest Statement

The authors declare that the research was conducted in the absence of any commercial or financial relationships that could be construed as a potential conflict of interest.
